# Nicotine Decreases Nerve Regeneration and Pain Behaviors via PTEN and Downstream Inflammation-Related Pathway in Two Rat Nerve Injury Models

**DOI:** 10.1523/ENEURO.0185-23.2023

**Published:** 2023-09-04

**Authors:** Yehong Fang, Tingkai Zhang, Ling Li, Shanshan Chen, Liangliang Wang, Jinsong Tang, Yanhui Liao

**Affiliations:** Department of Psychiatry, Sir Run Run Shaw Hospital, Zhejiang University School of Medicine, Hangzhou, Zhejiang 310016, People’s Republic of China

**Keywords:** nicotine, nerve regeneration, neuropathic pain, α7-nAChR, PTEN

## Abstract

Neuropathic pain is stubborn and associated with the peripheral nerve regeneration process. Nicotine has been found to reduce pain, but whether it is involved in the regulation of nerve regeneration and the underlying mechanism are unknown. In this study, we examined the mechanical allodynia thermal hyperalgesia together with the peripheral nerve regeneration after nicotine exposure in two rat neuropathic pain models. In the spinal nerve ligation model, in which anatomic nerve regeneration can be easily observed, nicotine reduced anatomic measures of regeneration as well as expression of regeneration marker growth-associated protein 43 (GAP43). In the tibial nerve crush model, nicotine treatment significantly suppressed GAP43 expression and functional reinnervation as measured by myelinated action potential and electromyography of gastrocnemius. In both models, nicotine treatment reduced macrophage density in the sensory ganglia and peripheral nerve. These effects of nicotine were reversed by the selective α7 nicotinic acetylcholine receptor (nAChR) blocker methyllycaconitine. In addition, nicotine significantly elevated expression of PTEN (the phosphatase and tensin homolog deleted on chromosome 10), a key player in both regeneration and pain. Pharmacological interference of PTEN could regulate GAP43 expression, pain-related behaviors, and macrophage infiltration in a nicotine-treated nerve crush model. Our results reveal that nicotine and its α7-nAChR regulate both peripheral nerve regeneration process and pain though PTEN and the downstream inflammation-related pathway.

## Significance Statement

The active peripheral nerve regeneration process is closely related to neuropathic pain. Nicotine has demonstrated pain relief properties, but whether nicotine is involved in peripheral nerve regeneration and whether this is related to its analgesic effect are unknown. In two rat nerve injury models, nicotine exposure not only reduced pain behaviors but also inhibited the nerve regeneration process in an α7 nicotinic acetylcholine receptor-dependent manner. In addition, nicotine significantly elevated the expression of PTEN, a phosphatase and tensin homolog deleted on chromosome 10, and pharmacological interference with PTEN reversed the inhibitory effect of nicotine on nerve regeneration. These findings provide a novel mechanistic understanding of the peripheral effect of nicotine on both peripheral nerve regeneration and pain.

## Introduction

In the clinic, tobacco smoking and pain frequently co-occur because of the positive feedback loop between them. Indeed, smokers with pain are more likely to use E-cigarettes and other nicotine products (Powers et al., 2020). However, accumulating research also indicates that sustained tobacco smoking alleviates pain and, in turn, that smoking cessation may exacerbate pain reactivity (Nakajima and Al'Absi, 2014; Baiamonte et al., 2016; Ditre et al., 2021). Subsequent animal studies verify that exposure to nicotine or systemic administration of nicotine has consistent antinociceptive effects in different rodent models (for review, see Shi et al., 2010). Furthermore, intranasal or transdermal nicotine has been explored as an adjunctive treatment for postoperative pain (Matthews et al., 2016).

Neuronal nicotinic acetylcholine receptors (nAChRs), the molecular targets of nicotine, and nicotinic cholinergic mechanisms contribute to dysfunctions such as schizophrenia, Parkinson’s disease, autism, Alzheimer’s disease (AD), and addiction (Dani and Bertrand, 2007). Drugs targeting nAChRs or ACh have curative effects on these diseases such as acetylcholinesterase inhibitors galantamine (Reminyl) for AD and the α4β2 partial agonist varenicline for smoking cessation. In addition, nAChRs also have been found to be involved in pain relief. Research by Teng et al. (2019) revealed that nicotine attenuates osteoarthritis pain via the peripheral α7 nAChR. Another study showed that antagonists of α9-containing nAChRs are analgesic in animal models of neuropathic pain (Hone et al., 2018). The evidence supports the idea that nicotine and nAChRs play vital roles in relieving pain.

Peripheral nerves are capable of regeneration and, when injury occurs, may cause neuropathic pain in the peripheral nervous system (PNS). Macrophage action, chemokine CCL2 (C-C motif ligand 2), and neuroinflammation have a positive effect in regeneration (Zigmond and Echevarria, 2019). Although numerous molecules (mainly cytokines and trophic factors) have been implicated in both nerve regeneration and pain, few studies had focused on the relationship between nerve regeneration and pain until the report by Xie et al. (2017). They found that active nerve regeneration with failed target reinnervation may be the origin of neuropathic pain. As nicotine and nAChR ligands inhibit macrophage action and neuroinflammation (Godin et al., 2020), we wonder whether nicotine and peripheral nAChRs play a role in reducing nerve regeneration and relieving neuropathic pain.

The phosphatase and tensin homolog deleted on chromosome 10 (PTEN) has been studied extensively in cancers and neurologic and psychiatric disorders (Chang et al., 2007; Wang et al., 2015). In addition, the role of PTEN in neuropathic pain has been reported (Huang et al., 2015). Our previous study has also found that spinal astrocytic PTEN plays a beneficial role in pain by regulating cholesterol biosynthesis (Fang et al., 2022). Moreover, studies revealed concurrent activation of PTEN/mTOR (mammalian target of rapamycin) and STAT3 pathways as a key for sustaining long-distance axon regeneration in the adult CNS (Sun et al., 2011). These results indicate PTEN as key player both in nerve regeneration and pain.

In this study, we examined the analgesic effect of nicotine in two rat neuropathic pain models. Then, we reported that nicotine administration reduced peripheral nerve functional and anatomic regeneration in an α7 nAChR-dependent manner. One molecule that was downregulated by nerve crush and reversed after intraperitoneal nicotine injection was PTEN. We found that inhibiting PTEN function in dorsal root ganglia (DRGs) could mimic many of the behavioral and biochemical effects of nicotine in the tibial crush model. The macrophage density in the sensory ganglia and peripheral nerve were also regulated by nicotine and PTEN. This research sheds light on the dual influence of nicotine on nerve regeneration and pain, providing a novel mechanistic understanding of its peripheral effects.

## Materials and Methods

### Animals and surgery

Adult Sprague Dawley rats (female or male; weight range, 180–220 g) were purchased from the SPF (Beijing) Biotechnology Co., Ltd. Rats were kept in the Laboratory Animal Center of the Sir Run Run Shaw Hospital, in a temperature-controlled and humidity-controlled environment under a controlled diurnal 12 h light/dark cycle with free access to food and water. All animal procedures performed in this study were reviewed and approved by the Institutional Animal Care and Use Committee of the Zhejiang University School of Medicine and were conducted in accordance with the guidelines of the International Association for the Study of Pain.

For the rat spinal nerve ligation (SNL) model (*n* = 56), procedures were produced according to the description by Ho Kim and Mo Chung (1992) and our previous study (Chen et al., 2019). Rats were anesthetized with sodium pentobarbital (50 mg/kg, i.p.; Sigma-Aldrich). After dissection of L5 and L4 transverse processes and isolation from the overlying muscles, the ventral ramus of the L5 spinal nerve was exposed. Then, the L5 spinal nerve was tightly ligated with 6–0 silk 2–3 mm distal to the ganglion and then cut 1 mm distal to the suture. The muscle and skin incisions were closed in layers. For the rat tibial nerve crush model (*n* = 70), the procedures were modified from the spared nerve injury (SNI) model (Decosterd and Woolf, 2000). Briefly, under anestheisa by isoflurane, the sciatic nerve and its three terminal branches (the sural and common peroneal and tibial nerves) were exposed by incising the right thigh skin and blunt dissecting through the biceps femoris muscle. The tibial nerve was then crushed 2–3 mm distal to the trifurcation for 30 s by a small arterial microclip with smooth protective pads formed by silicon tubing placed over the blades. At the end of this procedure, the tibial nerve was completely flattened and transparent. The incision was closed in layers. For sham operations, the surgery was performed only to expose the L5 spinal nerve or tibial nerve but without ligation and crush.

### Drugs and administration

Nicotine, methyllycaconitine (MLA), a selective α7 nAChR blocker (Gowayed et al., 2019; Papke and Horenstein, 2021), and a potent PTEN protector (Lee et al., 2019), indole-3-carbinol (I3C), were purchased from Sigma-Aldrich. The inhibitor of PTEN, SF1670, was purchased from Selleckchem. SF1670 and I3C were dissolved in dimethylsulfoxide (DMSO) first and diluted in saline solution, while nicotine and MLA were dissolved in saline solution directly. Rats had already received intraperitoneal injections of nicotine at 1 mg/kg and MLA at 1 mg/kg once daily for 1 week before surgery and continued receiving intraperitoneal injections of 1 mg/kg nicotine or MLA in sterile saline once daily for 3 week (Teng et al., 2019), whereas matched control rats received an equal volume of intraperitoneal saline. SF1670 (0.3 mg/kg; Li et al., 2011) and I3C (2 mg/kg; Lee et al., 2019) were injected intraperitoneally in nicotine-treated rats simultaneously until the date of surgery to observe the nociceptive effect of PTEN deficiency and analgesic effects for the prevention of PTEN degradation, respectively.

### Behavioral testing

Rats were acclimatized to the testing environment for 3 consecutive days before baseline testing and were tested for mechanical and thermal hyperalgesia in a blind manner to avoid potential bias. Before each test, habituation was performed for 30 min, as described in our previous studies (Fang et al., 2022). The region tested in the SNL model was centrally located and within the L4 dermatome, while the test site was moved somewhat more laterally to the sural nerve territory in the tibial nerve crush model. For the Hargreaves test (Hargreaves et al., 1988), a thermal stimulator (BME-410C Plantar Test Apparatus) was used. Rats (*n* = 84) were placed individually in a Plexiglas enclosure (10 × 20 × 20 cm) on top of a 2-mm-thick glass plate, and the plantar surface was exposed to a beam of radiant heat from the stimulator. The cutoff time was 20 s, and the test time interval between the tests of two feet was 5 min. The average of three recorded test times was considered the paw withdrawal thermal latency (s). For the von Frey test, rats (*n* = 84) were placed in a Plexiglas chamber (10 × 20 × 20 cm) placed on a wire grid floor, and the plantar surface of the hindpaw was stimulated using an electronic von Frey anesthesiometer (Electronic von Frey 2390, IITC Life Science). The maximum force (*g*) was automatically recorded, and the average of three successive force (*g*) readings was used as the paw withdrawal mechanical threshold (*g*).

### *In vivo* electrophysiological recording

An *in vivo* fiber recording method (Zhu et al., 2022) was used to estimate functional regeneration of myelinated action potential through the tibial crush site. On day 10 after tibial crush treated with saline, nicotine, or nicotine plus MLA, rats (*n* = 18) were anesthetized with sodium pentobarbital (50 mg/kg) with additional intravenous boluses of pentobarbital as needed and put on a warm blanket to keep the body temperature close to 37°C. The tibial nerve around both the injury site and sciatic nerve were exposed. Skin flaps were produced to form pools filled with warm light mineral oil to isolate the tibial nerve from surrounding nerves. For recording compound action potentials (CAPs), a 1-mm-diameter silver wire was inserted into the sciatic nerve transversely to serve as the recording electrode and a bipolar stimulating cuff electrode was placed distal to the crush site or proximal to the crush site, respectively, as a stimulating electrode. For recording evoked electromyography (EMG), a pair of needle recording electrodes was inserted into the gastrocnemius, which is innervated by the tibial nerve. The stimulating electrodes were placed proximal or distal to the crush site. Both CAP and EMG were elicited with 0.5 and 1.0 mA 1.0 ms stimuli, and were recorded by a low-noise differential amplifier and analyzed using Spike 2 software (Cambridge Electronic Design). The fast peak (Aα and Aβ fiber) of the CAP (average value from three stimuli per condition) and the peak of EMG were measured, and the ratio of distal to proximal amplitude was used as a measure of the fraction of fibers that failed to functionally regenerate through the crush site.

### Nerve and DRG microscopy

To measure anatomic nerve regeneration, the following two methods were used: (1) images of regenerated spinal nerves after 21 d of SNL surgery were obtained after the dissection of perfused rats, using a ZOOM-90 6.3 megapixel camera inserted into the eyepiece of a dissecting microscope; and (2) FAST Dil oil (5 mg/ml in DMSO; catalog #D3899, Thermo Fisher Scientific) was injected into the paw subcutaneously using 31 gauge insulin syringes after the surgery of SNL. At the end of behavior testing, sections (13 μm) of L5 DRGs and longitudinal sections (40 μm) of proximal L5 spinal nerves were obtained to determine whether Dil had been transported back to the ligated L5 DRG. Costaining of Dil and a subtype of neuronal markers was then conducted (see immunohistochemistry details below) to show a clear Dil-positive neurons.

### Nerve and DRG immunohistochemistry

Tissue for immunohistochemistry is from rats after 28 d of SNL and 4 d after tibial nerve crush. Rats were deeply anesthetized with sodium pentobarbital and transcardially perfused with 0.1 m PBS followed by 4% paraformaldehyde in 0.01 m PBS for 30 min. The L3–L5 DRGs in both models, L5 spinal nerve in SNL model, and tibial nerve in crush model were isolated, postfixed, and cut into sections for immunohistochemistry as described previously (Liu et al., 2017; Jiang et al., 2019). DRG sections were cut at 13 μm while tibial nerve longitudinal sections were cut at 40 μm. The following primary antibodies were used: growth-associated protein 43 (GAP43; 1:400; rabbit; catalog #ab16053, Abcam); Iba-1 (1:500; goat; catalog #ab5076, Abcam); NF200 (1:400; mouse; catalog #60331-1-1g, Proteintech); NeuN (1:400; mouse; catalog #ab104224, Abcam); calcitonin gene-related peptide (CGRP; 1:400; rabbit; catalog #14959T, Cell Signaling Technology); TRPV1 (1:400; guinea pig; catalog #ab10295, Abcam); PTEN (1:400; rabbit; catalog #10005059, Cayman Chemical); and PGP9.5 (1:400; guinea pig; catalog #ab10410, Abcam). Sections were then incubated with the proper secondary antibodies (Jackson ImmunoResearch) for 1 h at room temperature. Alexa Fluor 594-conjugated IB4 (1:400; Thermo Fisher Scientific) was added as a secondary antibody. Negative control followed the same steps without primary antibodies. The stained sections were examined using a laser confocal microscopic imaging system (FV1000 and FluoView software, Olympus). Images from five to eight sections of each DRG or nerve then were analyzed with ImageJ software for quantification. The signal intensity of a selected area in each channel was summed up. For GAP43 staining, both the neuronal cell bodies and areas predominantly containing axons were analyzed to get the intensity of the signal.

### Western blotting

After transcardial perfusion with PBS, the DRGs, L5 spinal nerve, and peripheral tibial nerve were harvested and homogenized in lysis buffer (CWBio) containing a protease inhibitor cocktail and a phosphate inhibitor cocktail (CWBio). After measuring protein concentrations using a Pierce BCA Protein Assay Kit (Thermo Fisher Scientific), the homogenates (20 μg total protein) were separated on 12% SDS–PAGE gels and transferred to polyvinylidene fluoride membranes. After blocking with 5% skim milk in TBST (Tris-buffered saline wash buffer with Tween 20), the membrane was incubated overnight at 4°C with primary antibodies. The following primary antibodies were used: GAP43 (1:1000; rabbit; catalog #ab16053, Abcam); Iba-1 (1:100; goat; catalog #ab5076, Abcam); PTEN (1:1000; rabbit; catalog #ab170941, Abcam); and GAPDH (1:3000; rabbit; catalog #110016, BBI Life Sciences). Following incubation with an HRP-conjugated secondary antibody for 30 min at room temperature, bands were detected using an EECL Kit and scanned by the ChemiDoc XRS System (BIO-RAD). The intensity of the selected bands was analyzed using ImageJ software.

### Statistical analysis

SPSS software (version 24.0) was used for statistical analysis. Data values are presented as the mean ± SEM. The one-way ANOVA with Tukey’s post-test was used in Western blotting and immunohistochemistry experiments for comparing differences among three or more groups. Two-way ANOVA followed by the Bonferroni’s *post hoc* test was used to determine significant differences in behavioral tests. Two-sided tests were used throughout. A statistically significant difference was defined as a *p* value < 0.05.

### Data availability

There are no data, software, databases, or application/tools available apart from those reported in the present study. All data are provided in the article.

## Results

### Nicotine alleviates pain behavior in SNL rats in a α7-nAChR dependent manner

**Figure 1.**
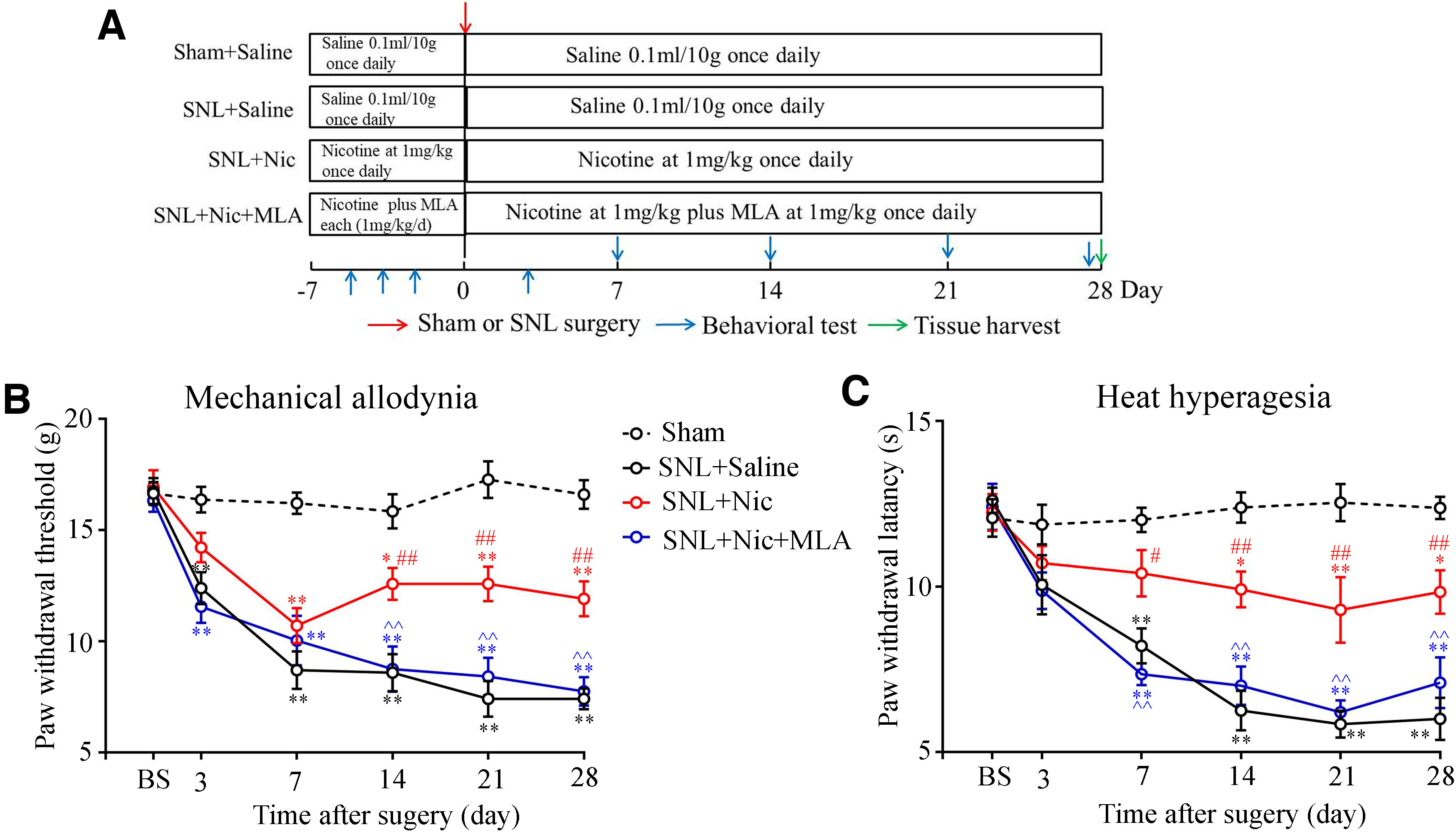
Nicotine alleviates mechanical allodynia and thermal hyperalgesia in SNL rats in a α7-nAChR-dependent manner. ***A***, The timeline shows nicotine, MLA and saline injection date, and the surgery and tissue harvest date. ***B***, ***C***, Intraperitoneal injection of nicotine significantly reduced mechanical allodynia (***B***) and thermal hyperalgesia (***C***) from 14 to 28 d after surgery compared with that of intraperitoneal injection of saline and MLA (1 mg/kg) reversed the analgesic effect (*n* = 6–8/group). *p <* *0.05, **p <* *0.01, versus the sham group; #*p *<* *0.05, ##*p *<* *0.01, versus the SNL plus saline group; ^^*p *<* *0.01, versus the SNL plus nicotine (1 mg/kg) group.

Next, we confirmed the analgesic effect of nicotine in animal models. The SNL model is a commonly used neuropathic pain model in which we confirm the analgesic effect of nicotine. After SNL, pronounced mechanical allodynia and heat hyperalgesia occur on day 3 and are sustained for many weeks (Ho Kim and Mo Chung, 1992). The nicotine was intraperitoneally injected into naive rats 7 d before surgery and was continued with a daily injection at a dose of 1 mg/kg. We checked the time course of pain behavior 3, 7, 14, 21, and 28 d after surgery (Fig. 1*A*, experimental timeline). The intraperitoneal administration of nicotine significantly reduced mechanical allodynia and thermal hyperalgesia from 14 to 28 d after surgery compared with intraperitoneal injection of saline (*p *<* *0.01, vs saline injection group; Fig. 1*B*,*C*). In addition, MLA (1 mg/kg), the selective α7 nAChR inhibitor, was able to reverse the analgesic effect of nicotine in the SNL model (Fig. 1*B*,*C*). These results indicate that nicotine alleviates neuropathic pain mainly through activating α7 nAChR.

### Nicotine reduces anatomic measurement of regeneration in SNL rats

**Figure 2.**
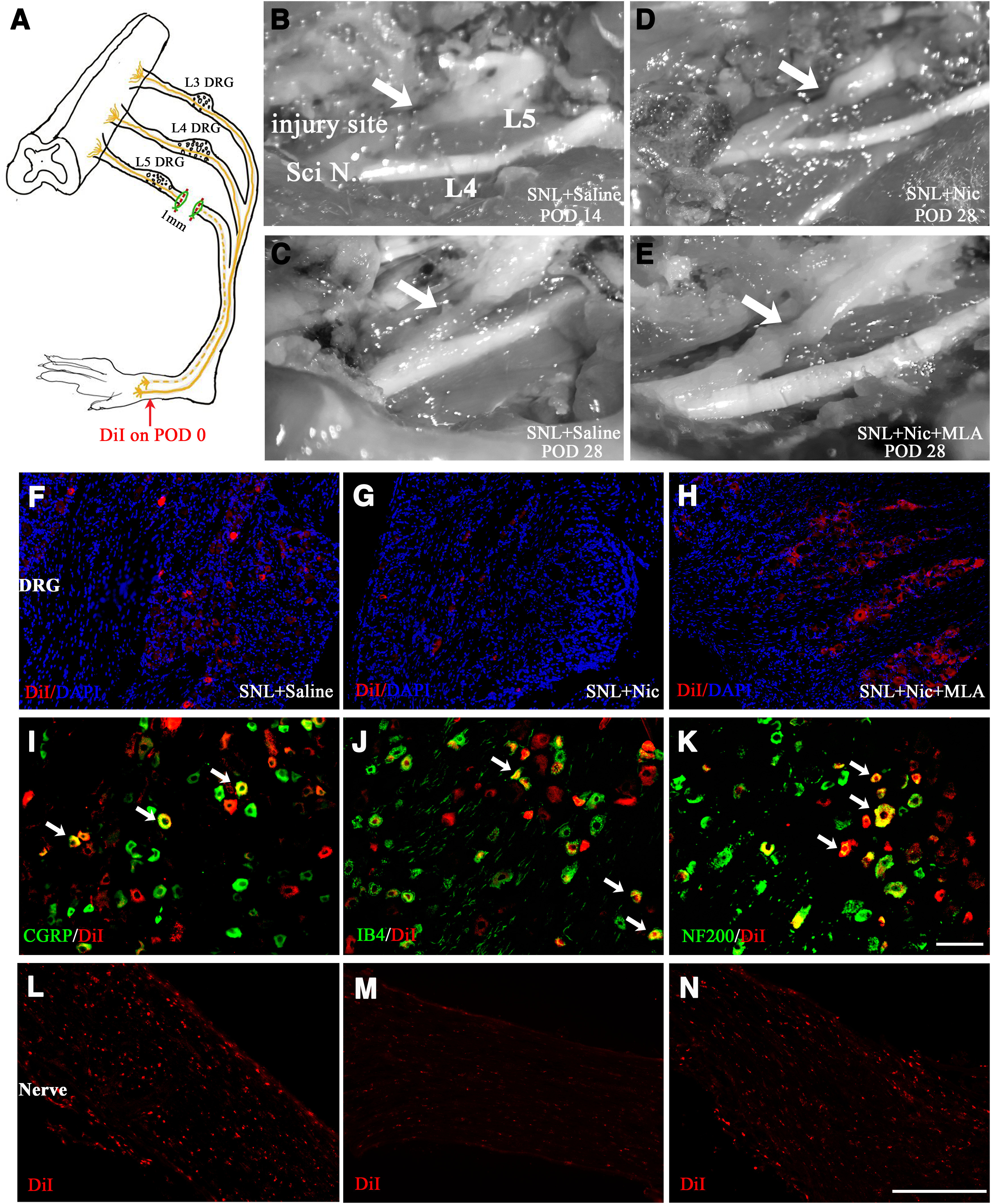
Nicotine inhibits L5 spinal nerve regeneration in the SNL model. ***A***, Schematic of an SNL model and the cut site of the L5 spinal nerve. The red arrow shows the injection site of Dil; Dil was injected just after the L5 nerve transection. ***B–E***, Microscope images demonstrating the regenerated L5 nerve (arrows) distal to the injury site observed on day 14 in saline group and on day 28 in the saline group, nicotine group, and nicotine plus MLA group. ***F–H***, Representative images of L5 DRG showing Dil transported from the hindpaw on day 28 in the saline group (***F***), nicotine group (***G***), and nicotine plus MLA group (***H***). ***I–K***, Double immunofluorescence labeling of Dil with CGRP (***I***), IB4 (***J***), and NF200 (***K***) in the L5 dorsal horn. Scale bar, 50 μm. ***L–N***, Representative images of L5 spinal nerve proximal to the injury site showing Dil transported from the hindpaw on day 28 in the saline group (***L***), nicotine group (***M***), and nicotine plus MLA group (***N***). Scale bar, 100 μm.

L5 spinal nerve ligation previously supposed could not regenerate or reinnervate, and the evoked pain behaviors are deemed to be mediated only by the intact neurons in the L4 DRG. However, in our previous study, we also found ectopic firing of C-neurons and even spontaneous discharges in L5 DRG (Chen et al., 2019). Moreover, direct evidence given by Xie et al. (2017) has shown that the L5 nerve can regenerate into the original sciatic nerve and reconstruct function. Here, similar to the report of Xie et al. (2017), we also observed the nerve regeneration beginning 2 weeks after the initial SNL surgery, and the new nerve regeneration site became white, more like the normal nerve after 28 d (Fig. 2*A–C*). Nicotine administration inhibited L5 spinal nerve regeneration, whereas the application of both MLA and nicotine seems to have no effect on nerve regeneration compared with that after the intraperitoneal injection of saline (Fig. 2*D*,*E*).

Additionally, the neuronal tracer Dil was injected into the ipsilateral hindpaw simultaneously with the SNL surgery to determine whether the L5 nerve regenerates after ligation (Fig. 2*A*). At the end of pain behavior testing, Dil was observed both in L5 DRG neurons and proximal L5 spinal nerves (Fig. 2*F*–*H*,*L*–*N*). The further costaining of Dil and neuronal markers showed that Dil could be observed in both NF200-positive myelinated neurons and CGRP-positive and IB4-positive unmyelinated neurons (Fig. 2*I*–*K*). Nicotine administration blocked Dil transmitting to the L5 DRGs, which indicated the effect of nicotine on preventing regeneration and could be reversed by MLA injection (Fig. 2*F*–*H*,*L*–*N*).

### Nicotine reduces GAP43 expression in DRG and spinal nerve after L5 nerve ligation

**Figure 3.**
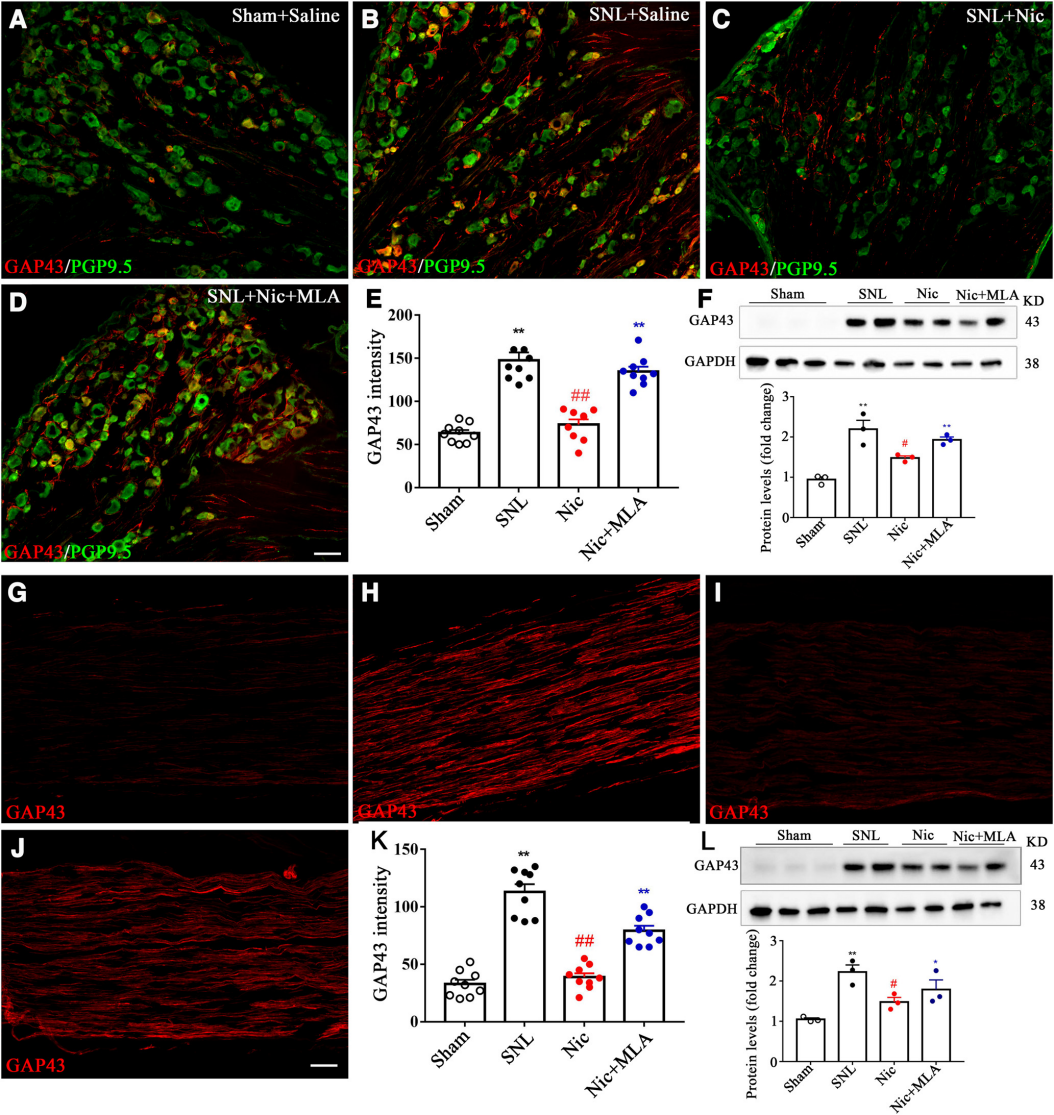
Nicotine reduces GAP43 expression in DRG and spinal nerve after L5 nerve ligation. ***A–D***, Representative immunofluorescence images of L5 DRG showing GAP43 (red) and neuronal marker PGP9.5 (green) expression in the sham group (***A***), SNL + saline group (***B***), SNL + nicotine group (***C***), and SNL + nicotine + MLA group (***D***). ***E***, Immunofluorescence intensity analysis shows that the immunoreactivity of GAP43 in the L5 DRG was reduced at postoperative day 28 in the nicotine group and reversed in the nicotine plus MLA group. *n* = 8 sections from 3 rats per group. ***F***, Representative Western blots and quantification of GAP43 in the above four groups. ***G–J***, Representative immunofluorescence images of L5 spinal nerve showing GAP43 expression in the sham group (***G***), SNL + saline group (***H***), SNL + nicotine group (***I***), and SNL + nicotine + MLA group (***J***). ***K***, Immunofluorescence intensity analysis shows that the immunoreactivity of GAP43 in the L5 spinal was reduced at postoperative day 28 in the nicotine group and reversed in nicotine plus MLA group. *n* = 9 sections from 3 rats per group. ***L***, Representative Western blots and quantification of GAP43 in L5 spinal nerve in the above four groups. *n* = 3. *Compared with the sham group; #compared with the SNL group. Scale bar, 50 μm.

**Figure 4.**
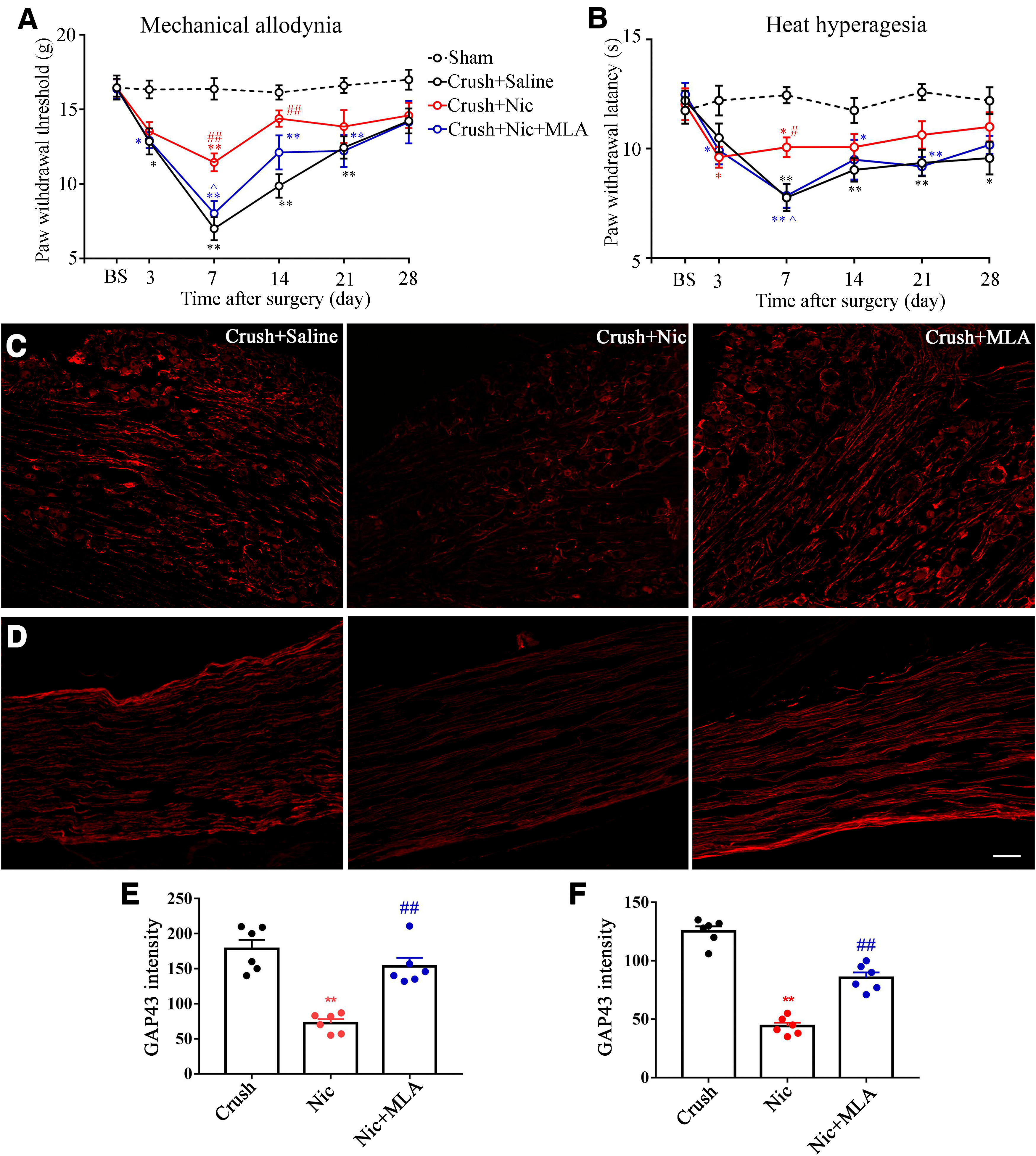
Nicotine alleviates pain behaviors and reduced GAP43 expression in the nerve crush model. ***A***, ***B***, Nicotine injection alleviated mechanical allodynia (***A***) and thermal hyperalgesia (***B***) in comparison with vehicle (saline solution) injection and MLA reversed this effect. *n* = 7/group. ***C***, ***D***, DRG sections from cellular regions (***C***) or axonal regions (***D***) stained for GAP43 from crush + saline group, crush + nicotine group, and crush + nicotine + MLA group. Scale bar, 100 μm. ***E***, ***F***, Summary data of GAP43 intensity from cellular (***E***) and axonal (***F***) regions. **p <* *0.01, versus the crush plus saline group; ##*p *<* *0.01, versus the crush plus nicotine group; ^^*p *<* *0.01, versus the SNL plus nicotine (1 mg/kg) group.

To examine the effect of nicotine and α7 nAChR on regeneration, GAP43 expression was examined by immunofluorescence analysis and Western blotting. GAP43 is a protein kinase C-activated phosphoprotein and often serves as a marker in axonal plasticity and regeneration (Hung et al., 2016). The basal lever of GAP43 expression was low in rats with sham surgery and markedly increased (a nearly threefold change) at postoperative day 28 (POD28) in SNL rats (Fig. 3*A*,*B*). Nicotine significantly reduced the GAP43 expression level, and MLA reversed that expression (Fig. 3*C*–*E*, summary data). The following Western blotting and quantification results further confirmed the effect of nicotine and MLA on GAP43 expression (Fig. 3*F*). In addition, marked upregulation was also observed in the axon just distal to the L5 DRG and proximal to the ligation site after SNL surgery. The GAP43 expression in the axon after nicotine and MLA administration was changed similar to that in the DRG, as in both the immunofluorescence and Western blot analyses shown in Figure 4*G*–*L*.

### Nicotine suppresses neuropathic pain and GAP43 expression after tibial nerve crush

Nerve crush model was commonly used as an experimental model in modeling nerve injury and regeneration (Bridge et al., 1994), such as optic nerve crush in CNS and sciatic nerve crush in PNS. Here, the tibial nerve crush model was used to reconfirm the effect of nicotine on nerve regeneration and neuropathic pain. In contrast to the SNL model, tibial nerve crush significantly induced thermal hyperalgesia and mechanical allodynia from 3 d and peaked at 7 d after surgery compared with sham operation (*p *<* *0.05 or 0.01; Fig. 4*A*,*B*). The intraperitoneal administration of nicotine significantly reduced mechanical allodynia and thermal hyperalgesia from 7 to 14 d after surgery compared with intraperitoneal injection of saline (*p *<* *0.01, vs saline injection group; Fig. 4*A*,*B*). In addition, MLA (1 mg/kg) could also reverse the analgesic effect of nicotine in the nerve crush model (Fig. 4*A*,*B*).

We also examined the effect of nerve crush on the expression of GAP43. As shown in Figure 4*C*–*F*, the GAP43-positive neurons and GAP43-positive axons coursing between the cell bodies in the DRG and GAP43-positive axon proximal to the crush site were observed 4 d after nerve crush. Nicotine reduced the intensity of GAP43 expression and the further MLA administration significantly promoted the expression of GAP43 (a nearly twofold change compared with the nicotine injection group, *p *<* *0.01).

### Nicotine reduces functional measurement of regeneration

**Figure 5.**
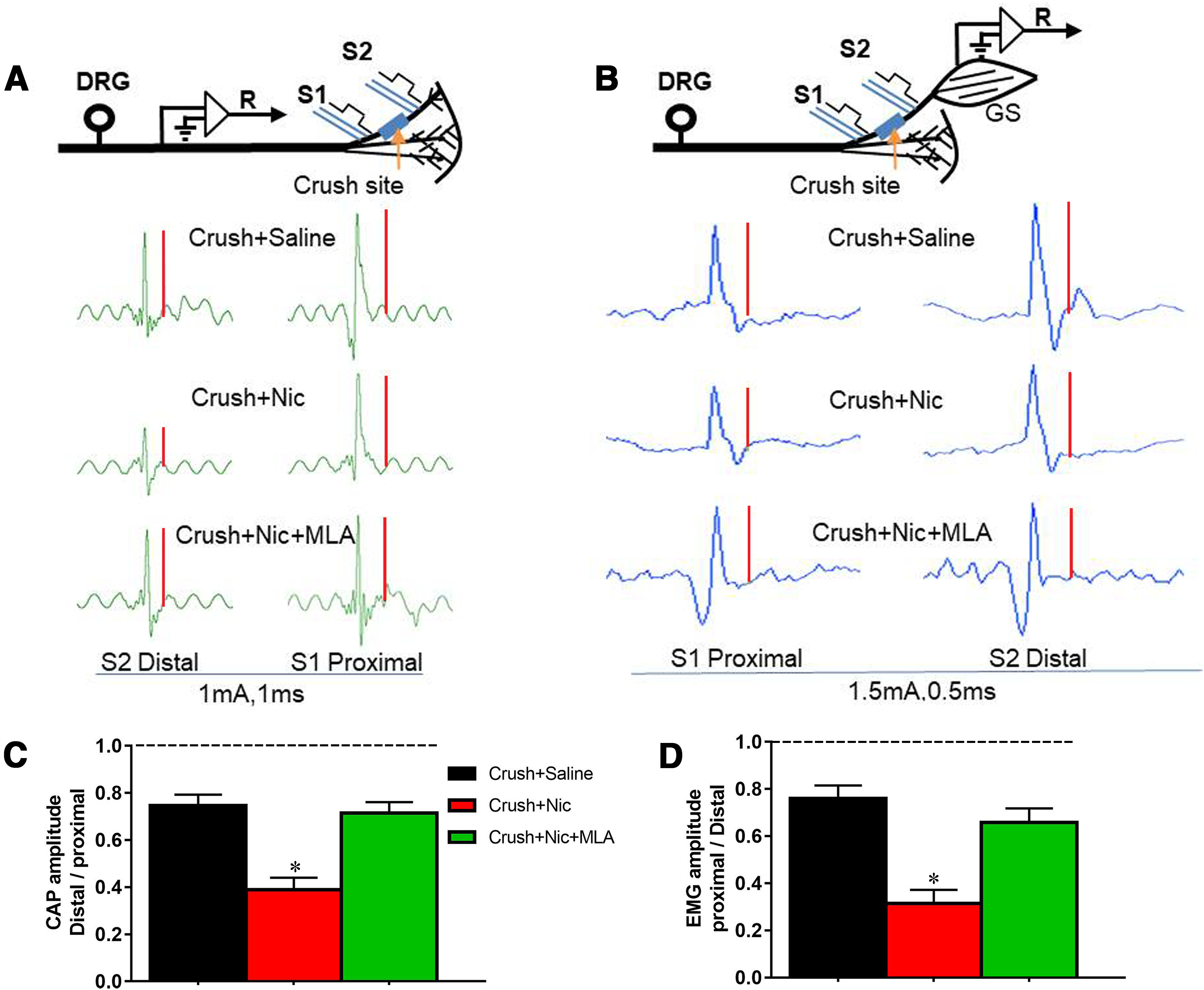
Effect of nicotine on functional measurement of regeneration. ***A***, The schematic and representative recording trace showing *in vivo* recording setup of CAPs 10 d after tibial crush injury in the saline injection, nicotine injection, and nicotine plus MLA injection groups. The CAPs were measured in response to stimulation distal to the injury site (S2) and then proximal to the crush site (S1). ***B***, The schematic and representative recording trace showing EMG recording of gastrocnemius setup 10 d after tibial crush injury in saline injection, nicotine injection, and nicotine plus MLA injection groups. The EMG was measured in response to stimulation proximal to the crush site (S1) and distal to the injury site (S2). ***C***, ***D***, The ratios of early component of CAPs (***C***) and EMG (***D***) evoked with distal and proximal stimuli were measured in the above three groups, respectively. A ratio value of 1 would be expected for a normal nerve, and 0 would be expected for no regeneration of nerve. *N* = 6 rats/group. *p <* *0.05.

The above examination of GAP43 expression in the tibial nerve crush model suggested that nicotine may reduce the nerve regeneration in an α7 nAChR-dependent manner. To determine whether functional regeneration was also reduced by nicotine, both in *vivo* fiber recording and EMG of gastrocnemius were conducted in crush plus saline, crush plus nicotine, and crush plus nicotine plus MLA rats. The recording electrode was placed on the sciatic nerve while the stimulating electrode was placed first on the tibial nerve distal to the nerve crush site and then moved to the tibial nerve proximal to the crush site. The CAP of the sciatic nerve was measured twice and the ratio of the peak of the A component in CAP from the distal stimulation to that of the proximal stimulation was analyzed as an indicator of functional regeneration (Fig. 5*A*,*B*). This ratio was significantly lower in rats that received nicotine and was reversed under nicotine plus MLA treatment (Fig. 5*A*,*C*).

The EMG of gastrocnemius innervated by tibial nerve was then recorded in the same three groups. This time the ratio of EMG from the proximal stimulation to that of the distal stimulation was analyzed. The quantification results showed that nicotine also reduced this ratio and MLA counteracted this ratio change tendency (Fig. 5*B*,*D*).

### Tibial nerve crush-induced decreases in PTEN and increases in microphage density are reversed by nicotine administration

**Figure 6.**
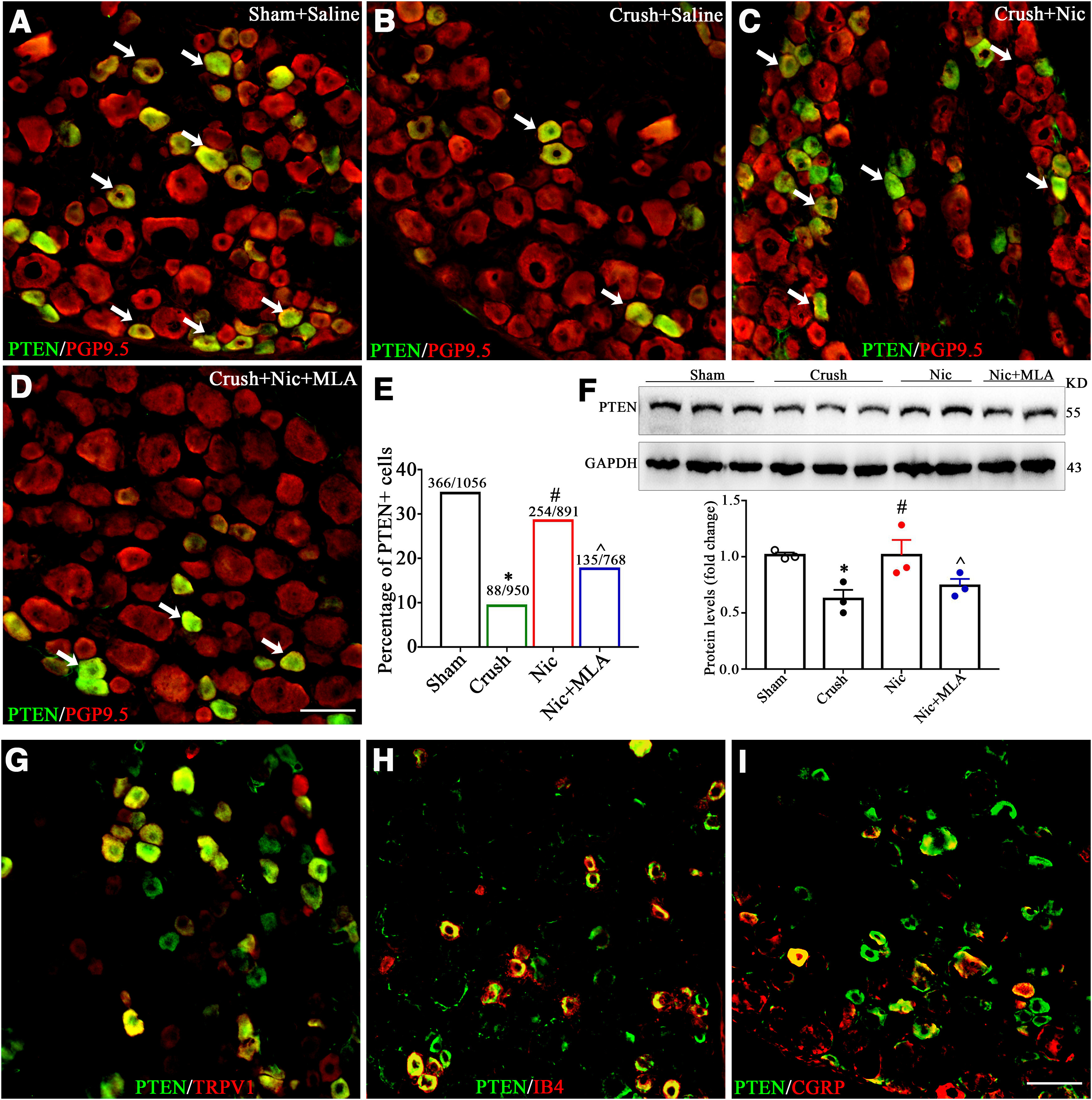
Nicotine increases PTEN expression in DRG nociceptive neurons. ***A–D***, Immunostaining shows PTEN in PGP9.5-positive neurons in sham plus saline, crush plus saline, crush plus nicotine, and crush plus nicotine plus MLA groups. Scale bar, 50 μm. ***E***, The percentage of PTEN**-**positive neurons was reduced in the crush plus saline group and reversed in the crush plus nicotine groups. *n* = 3. *p <* *0.05, versus the sham plus saline group; #*p *<* *0.05, versus the crush plus saline group; ^*p *<* *0.05, versus the crush plus nicotine group. ***F***, Western blot shows the effect of nicotine on PTEN expression. *n* = 3. *p <* *0.05, versus the sham plus saline group. ***G–I***, Double immunofluorescence staining of PTEN with nociceptive cell markers TRPV1, IB4, and CGRP. *n* = 3. Scale bar, 50 μm.

We previously have found that nerve injury induces astrocytic PTEN downregulation in rat spinal cord and that overexpression of PTEN attenuates neuropathic pain (Fang et al., 2022); but the PTEN in DRGs remains scantily understood. Here, we measured the immunohistochemistry of PTEN in DRGs and found that PTEN was expressed in sensory neurons. Compared with the sham group (34.7%), PTEN-positive neurons were significantly downregulated on POD28 (9.2%) crush rats in DRGs (Fig. 6*A*,*B*). Next, the effect of nicotine and MLA on PTEN expression was examined on POD28 crush rats. Intraperitoneal injection of nicotine upregulated PTEN expression, and MLA reversed that expression (Fig. 6*C–E*). The following Western blotting and quantification results further confirmed the effect of nicotine and MLA on PTEN expression (Fig. 6*F*). According to the double immunofluorescent staining results, PTEN was mainly distributed on sensory neurons and was costained with three nociceptive markers, including TRPV1, IB4, and CGRP (Fig. 6*G–I*). These data suggested that crush nerve injury induces the reduction of PTEN expression peripherally in DRGs and that the nicotine-mediated PTEN pathway may be involved in pain and regeneration.

**Figure 7.**
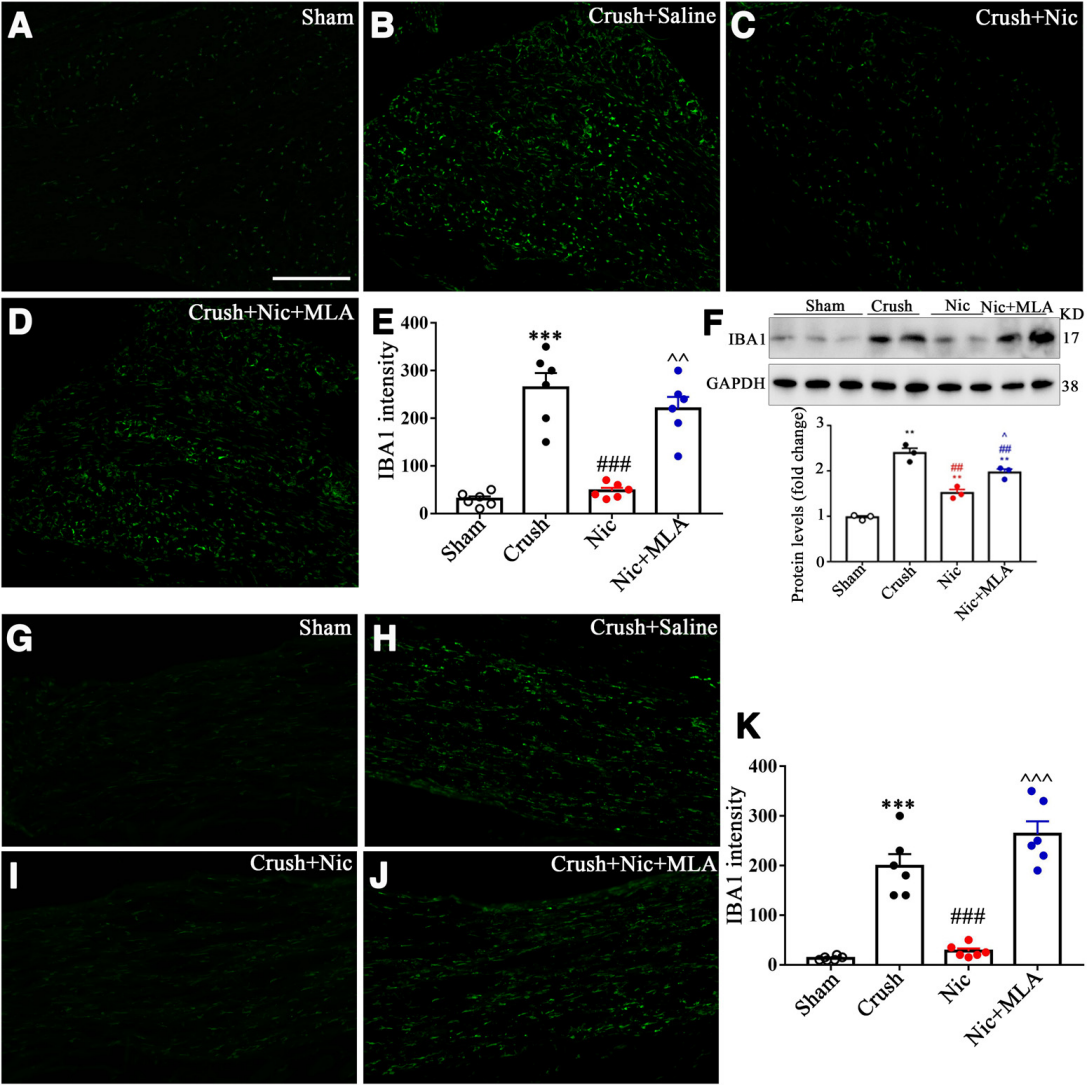
Nicotine administration reduces microphage density in DRG. ***A–D***, Immunostaining of IBA1 in the sham plus saline, crush plus saline, crush plus nicotine, and crush plus nicotine plus MLA groups in DRG. Scale bar, 50 μm. ***E***, The IBA1 intensity shows nicotine reduced microphage density and was reversed by MLA. *n* = 6. ***p <* *0.001, versus the sham plus saline group; ###*p *<* *0.001, versus the crush plus saline group; ^^*p *<* *0.01, versus the crush plus nicotine group. ***F***, Western blot shows the effect of nicotine on IBA1 expression. *n* = 3. ***G–J***, Immunostaining of IBA1 in the sham plus saline, crush plus saline, crush plus nicotine, and crush plus nicotine plus MLA groups in nerves. Scale bar, 50 μm. ***K***, The IBA1 intensity analysis. *n* = 6. ***p <* *0.001, versus the sham plus saline group; ###*p *<* *0.001, versus the crush plus saline group; ^^^*p *<* *0.001, versus the crush plus nicotine group.

Previous work (Piao et al., 2009) has found that nicotine had an obvious anti-inflammatory effect peripherally. Nicotine and α7-nAChR suppressed M1 macrophage polarization against inflammation (Han et al., 2021). Therefore, we next examined the effect of nicotine on microphage density using the pan-macrophage marker IBA1, in both DRGs and a distal nerve segment 4 d after crush. As shown in Figure 7, both immunoreactivity and Western blot of IBA1 that was increased in the tibial nerve crush model was reduced by nicotine and reversed by α7-nAChR blocker MLA, suggesting the effect of nicotine on immune homeostasis in peripheral DRGs and nerves.

### Administration of inhibitor of PTEN SF1670 reversed analgesic effect, GAP43 expression, and microphage density of nicotine

**Figure 8.**
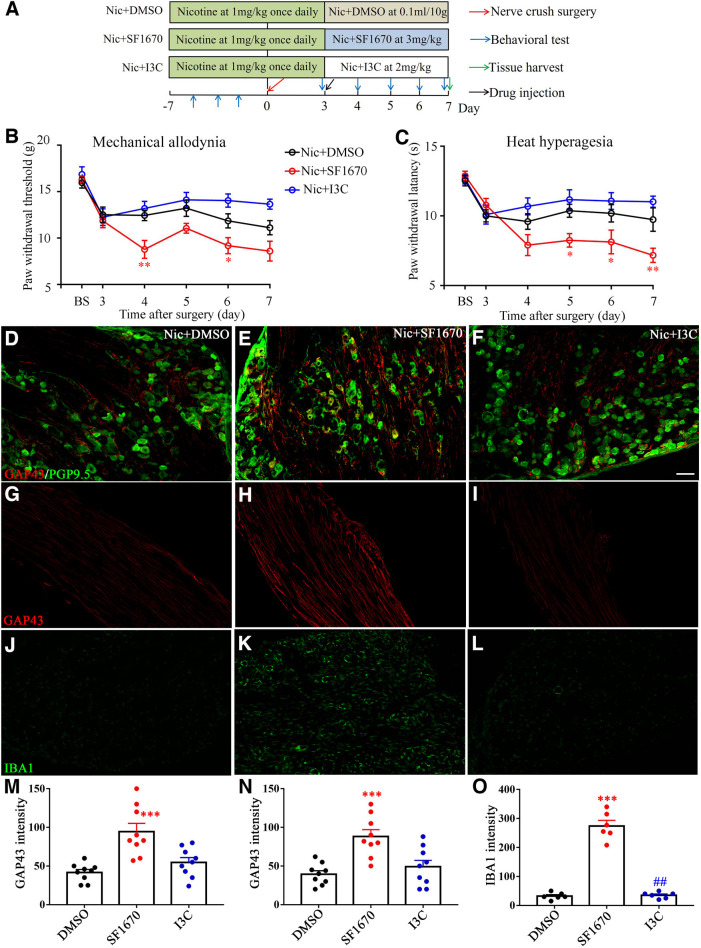
The PTEN inhibitor SF1670 negates analgesic effects of nicotine. ***A***, Diagram showing **the** nicotine, SF1670, and I3C injection dates, the surgery and tissue harvest dates, and the behavioral test timeline. ***B***, ***C***, Intraperitoneal injection of SF1670 significantly reduced mechanical allodynia (***B***) and thermal hyperalgesia (***C***) from 4 to 7 d after surgery compared with that of intraperitoneal injection of DMSO (*n* = 6–8/group). *p <* *0.05, **p <* *0.01, versus the nicotine plus SF1670 group. ***D–I***, DRG sections from cellular regions (***D–F***) or axonal regions (***G–I***) stained for GAP43 from nicotine + DMSO group, nicotine + SF1670 group, and nicotine + I3C group. Scale bar, 100 μm. ***J–L***, DRG sections from cellular regions stained for IBA1. ***M***, ***N***, Summary data of GAP43 intensity from cellular (***M***) and axonal (***N***) regions. ***O***, Summary data of GAP43 intensity from cellular regions ***p <* *0.001, versus the nicotine plus DMSO group; ##*p *<* *0.01, versus the nicotine plus SF1670 group.

To determine the role of PTEN on the analgesic effect of nicotine, the PTEN inhibitor SF1670 was injected intraperitoneally into the crush rats on POD3 and continued injecting until POD7 once daily (Fig. 8*A*). Then, we checked the time course of pain behavior once daily. The injection of SF1670 (100 μl, 3 mg/kg, i.p.) reversed the analgesic effect of nicotine, as shown in descending mechanical and thermal thresholds compared with that of intraperitoneal injection of DMSO (*p *<* *0.05 or *p *<* *0.01 vs DMSO injection group; Fig. 8*B*,*C*). In addition, the coantinociceptive effect of nicotine and I3C was also examined in nerve crush rats. I3C is a derivative of glucobrassicin in cruciferous vegetables, which prevents PTEN loss *in vivo* (Lee et al., 2019). After the injection of I3C (2 mg/kg, 100 μl) on day 3 after nerve crush (Fig. 8*A*), both mechanical allodynia and thermal hyperalgesia induced by nerve crush operation was alleviated but showed no significant difference compared with the nicotine injection group (*p* > .05; Fig. 8*B*,*C*).

The effect of PTEN on nerve regeneration was also examined by immunofluorescent staining of GAP43. As expected, SF1670 induced upregulated GAP43 expression in both DRG and distal nerve segment, as observed on day 7 after the nerve crush (*p *<* *0.001, vs DMSO group; Fig. 8*D–I*,*M*,*N*). This upregulation was reduced by I3C injection on day 3 after the nerve crush (Fig. 8*D–I*,*M*,*N*).

Next, the immunoreactivity of IBA1 was reduced by nicotine, and was increased with the PTEN inhibitor SF1670. The protector of PTEN I3C injection reduced the IBA1 signal in DRGs (Fig. 8*J–L*,*O*).

Together, these data indicate that PTEN plays an essential role in nicotine-related analgesia and inhibition of nerve regeneration.

## Discussion

Clinically, it seems intricate and disputable for understanding the interrelationship between nicotine intake and pain condition. Since the comorbidity of tobacco smoking and pain and the reciprocal model of the positive feedback loop (Kosiba et al., 2018), a collection of evidence supports the idea that nicotine may increase pain. The epidemiological evidence also shows that smoking is a risk factor for chronic pain (Shi et al., 2010). However, abundant empirical and epidemiological studies reveal that nicotine has analgesic properties, especially in individuals with mental disorders (Crocq, 2003; Ditre et al., 2016). Animal studies show consistent support for direct pain-inhibitory effects of nicotine, but a potential mechanism remains obscure. Here, we investigated the analgesic effect of nicotine and indicated that nicotine may alleviate the peripheral nerve regeneration process and pain through regulating PTEN and the downstream inflammation-related pathway.

Present results show that daily injection of nicotine reduced both mechanical and thermal pain intensity robustly in SNL and nerve crush models for 28 d. These data reflect the acute analgesic effect of nicotine on pain, especially neuropathic pain. It is worth noting that the analgesic effect was partial, because the von Frey threshold in two models was regained from low values (<8 g) in the saline group to 10–13 g in the nicotine injection group (as measured on day 14 after surgery), compared with the baseline level of nearly 17 g (Figs. 2*B*, 5*A*). A study in osteoarthritis pain concurred with this finding (Teng et al., 2019). As some studies point out that chronic exposure to nicotine may impair the sensitivity of nAChRs (Buisson and Bertrand, 2001), the long-tern effect of nicotine is questionable and should be verified in the future. Also the analgesic effect of nicotine on other types of pain, like inflammatory pain and cancer-induced pain, should be investigated.

How nicotine mitigates pain intensity and experience is currently not fully understood. Nicotine exerts its pharmacological function by interacting with the nAChR family. In peripheral, the α7 and α9α10 nAChRs are found expressed in DRGs and are vital for pain regulation and relieving (Shi et al., 2010; Papke and Horenstein, 2021; Zhou et al., 2022). Thus, we used the α7 nAChR blocker MLA to confirm its function on pain and found that MLA reversed the analgesic effect of nicotine. This result indicates that nicotine functions as a pain killer in a α7 nAChR-dependent manner.

Nerve regeneration per se is the normal physiological process for function recovery after nerve injury but may be the origin of neuropathic pain (Xie et al., 2017; Cranfill and Luo, 2022; Gangadharan et al., 2022). The active nerve regeneration, the miswiring or failed target reinnervation may cause neuropathic pain, and blocking regeneration in multiple ways relieves pain (Xie et al., 2017; Ritzel and Wu, 2023). To this end, we wonder whether nicotine also influences the nerve regeneration process. As reinnervation in the SNL model often results in neuroma formation (Toia et al., 2015), the SNL and tibial nerve crush models, in which the effective nerve regeneration can be observed, were used. To our surprise, nicotine inhibited the new nerve regeneration in an α7 nAChR-dependent manner, as shown in the anatomic images and Dil tracer results. The following immunofluorescence and Western blotting analysis of GAP43 (a molecule found strongly correlated with the nerve regeneration process; Mahar and Cavalli, 2018; Zhang et al., 2023) and functional regeneration measured by in *vivo* fiber recording and electromyography also showed the reduction regeneration after nicotine administration. Together, these results indicate that nicotine disrupted the nerve regeneration process, and this may partly explain the analgesic effect of nicotine. These results also give a reminder that smoking cessation is necessary for smokers after nerve injury.

Neuroimmune cross talk and macrophages have been uncovered that play a role in both pain and nerve regeneration (Domoto et al., 2021; Luo et al., 2021). Both the tissue-resident macrophages and peripheral nerve macrophages secrete a variety of mediators, such as high mobility group box 1, interleukin 1β (IL-1β), and IL-6, that regulate the excitability of primary afferents and neurons and contribute to the pathogenesis of inflammatory and neuropathic pain (Sekiguchi et al., 2018). We observed that nicotine reduced microphage density in both DRG and distal nerve, which indicated the effect of nicotine on immunity and inflammation. This result is consistent with the idea that nicotine is an anti-inflammation molecule in both the nervous system and the immune system (Zhang et al., 2022). As M2 phenotype macrophages have been found to promote regeneration and repair (Kwon et al., 2015), the nicotine-induced reduction of regeneration is harder to explain. One possible explanation is that regeneration may require an initial period of type 1 inflammation, and that the anti-inflammatory effect of nicotine weakens the nerve regeneration.

We also show that PTEN was reduced in DRG after tibial nerve crush and nicotine reversed this change. Moreover, the administration of the PTEN inhibitor SF1670 reversed the effect of nicotine on both neuropathic pain and regeneration. Studies have found that simultaneous deletion of PTEN and SOCS3 enables sustained axon regeneration in CNS (Sun et al., 2011) and the overexpression of astrocytic PTEN alleviates neuropathic pain (Fang et al., 2022). Combined with these findings, it is possible that PTEN is a downstream molecule involved in nicotine regulation of pain and regeneration, but the specific mechanism of how nicotine regulates PTEN needs to be further studied in the future.

In conclusion, our study provides evidence for a close association among nicotine, peripheral nerve regeneration, and neuropathic pain. Further mechanistic investigations demonstrate that nicotine inhibits peripheral nerve regeneration and pain through regulating PTEN and downstream anti-inflammatory pathways in an α7-nAChR-dependent manner. Insofar as there is a contradiction between the effect of nicotine on regeneration and pain, it is essential to understand whether it is possible to block the development of pain without inhibiting nerve regeneration, in a condition in which such regeneration is desirable at an early stage of nerve injury.

## Synthesis

Reviewing Editor: David Schoppik, New York University Decisions are customarily a result of the Reviewing Editor and the peer reviewers coming together and discussing their recommendations until a consensus is reached. When revisions are invited, a fact-based synthesis statement explaining their decision and outlining what is needed to prepare a revision will be listed below. The following reviewer(s) agreed to reveal their identity: Judith Strong.

The data are generally convincing and well-presented.

Our primary concern is that specificity of the reagents used and doses chosen needs to be better justified and/or cited. There are no references given for methyllycaconitine (MLA) as a selective α7 nAChR blocker. It is a long-used compound but some reference or review reference should be given, and the method of choosing the dose given or other justification for concluding it only works through this particular subunit should be given (or that conclusion toned down). Similarly, one of the references given for Indole-3-carbinol (I3C) as a PTEN “protector” (Choi et al., 2019) seems to be incorrect, this paper seems to be about cytochrome P450c17 and does not mention Indole-3-carbinol, at least by that name. The other cited reference (Qi et al., 2005) uses dietary application. Although the authors present data for a role for PTEN as a site of action for the I3C a quick literature search suggests this substance is likely to have multiple sites of action. This caveat should be discussed and the dose selected justified.

Minor suggestions and corrections:

Some minor English editing is needed throughout.

Lines 51-53 “Another study showed antagonists of 51 α9-containing nAChRs are analgesic in animal models of neuropathic pain(Hone et al., 2018). These evidences support nicotine and nAChRs as analgesic for pain” - This is confusing because if nAChRs are analgesic, antagonists should be pro-nociceptive. Perhaps move this citation to the discussion where there is a more complete discussion of the complexities surrounding nicotine and pain.

Why is the nicotine given for a full week before the pain model - is this to mimic the longer term exposure seen in smokers?

Somewhere it might be good to indicate that the fiber recording method (if I understand the details correctly) would not distinguish between fibers that failed to regenerate and those that regenerated but had not re-myelinated; the latter might still be visible in microscopy.

Why do there seem to be two spikes when the stimulus was placed distal to the injury (left side of figure 5). The green of the traces and the blue of the line showing the measured value may be too close in color, perhaps make them a different color or red like the proximal traces have.

line 248-249 if Bonferroni correction was used, then alpha should be less than 0.05. Please report appropriately.

line 273 epibiotic is not the right word. Episodic? Ectopic?

TBST line 232, spell out abbreviation

Line 303-304 “(Fig. 3C, 3D and 4E for summary data)” should be 3E

Throughout - western blot/blotting not bolt/bolting

Figure 6F, legend refers to significance symbols but they are not on the graph. Statistical method for the percentages of positive neurons needs to be added to the methods section (assumed it was chi square).

The methods section could use more details about how IHC signals were quantified for the summary data. In some figures (e.g. figure 3 E, K) the legend gives n = 3 rats per group but there seem to be >3 points in the scatter plots - are statistics being done on sections instead of the usual animal averages?

In Figure 8 and accompanying text - we assumed that the nicotine injections continued throughout the experiment, as in other experiments (otherwise one would need information about the time course for decay of the nicotine effect), but the graphic makes it look like the nicotine stopped at the day of surgery. It may be hard to indicate this without making Figure 8A too busy (perhaps say “+ DMSO...daily”? etc.?, but at any rate this should be clarified in the text describing that figure.
